# Pain and temperature processing in dementia: a clinical and neuroanatomical analysis

**DOI:** 10.1093/brain/awv276

**Published:** 2015-10-13

**Authors:** Phillip D. Fletcher, Laura E. Downey, Hannah L. Golden, Camilla N. Clark, Catherine F. Slattery, Ross W. Paterson, Jonathan D. Rohrer, Jonathan M. Schott, Martin N. Rossor, Jason D. Warren

**Affiliations:** Dementia Research Centre, UCL Institute of Neurology, University College London, London, UK

**Keywords:** pain, temperature, sensory, frontotemporal dementia, Alzheimer’s disease

## Abstract

Symptoms suggesting altered processing of pain and temperature have been described in dementia diseases and may contribute importantly to clinical phenotypes, particularly in the frontotemporal lobar degeneration spectrum, but the basis for these symptoms has not been characterized in detail. Here we analysed pain and temperature symptoms using a semi-structured caregiver questionnaire recording altered behavioural responsiveness to pain or temperature for a cohort of patients with frontotemporal lobar degeneration (*n = *58, 25 female, aged 52–84 years, representing the major clinical syndromes and representative pathogenic mutations in the *C9orf72* and *MAPT* genes) and a comparison cohort of patients with amnestic Alzheimer’s disease (*n = *20, eight female, aged 53–74 years). Neuroanatomical associations were assessed using blinded visual rating and voxel-based morphometry of patients’ brain magnetic resonance images. Certain syndromic signatures were identified: pain and temperature symptoms were particularly prevalent in behavioural variant frontotemporal dementia (71% of cases) and semantic dementia (65% of cases) and in association with *C9orf72* mutations (6/6 cases), but also developed in Alzheimer’s disease (45% of cases) and progressive non-fluent aphasia (25% of cases). While altered temperature responsiveness was more common than altered pain responsiveness across syndromes, blunted responsiveness to pain and temperature was particularly associated with behavioural variant frontotemporal dementia (40% of symptomatic cases) and heightened responsiveness with semantic dementia (73% of symptomatic cases) and Alzheimer’s disease (78% of symptomatic cases). In the voxel-based morphometry analysis of the frontotemporal lobar degeneration cohort, pain and temperature symptoms were associated with grey matter loss in a right-lateralized network including insula (*P < *0.05 corrected for multiple voxel-wise comparisons within the prespecified anatomical region of interest) and anterior temporal cortex (*P < *0.001 uncorrected over whole brain) previously implicated in processing homeostatic signals. Pain and temperature symptoms accompanying *C9orf72* mutations were specifically associated with posterior thalamic atrophy (*P < *0.05 corrected for multiple voxel-wise comparisons within the prespecified anatomical region of interest). Together the findings suggest candidate cognitive and neuroanatomical bases for these salient but under-appreciated phenotypic features of the dementias, with wider implications for the homeostatic pathophysiology and clinical management of neurodegenerative diseases.

## Abstract (precis)

Symptoms suggesting altered pain and temperature processing have been described in dementia diseases. Using a semi-structured caregiver questionnaire and MRI voxel-based morphometry in patients with frontotemporal degeneration or Alzheimer’s disease, Fletcher *et al.* show that these symptoms are underpinned by atrophy in a distributed thalamo-temporo-insular network implicated in somatosensory processing.

## Graphical abstract

**Figure:**
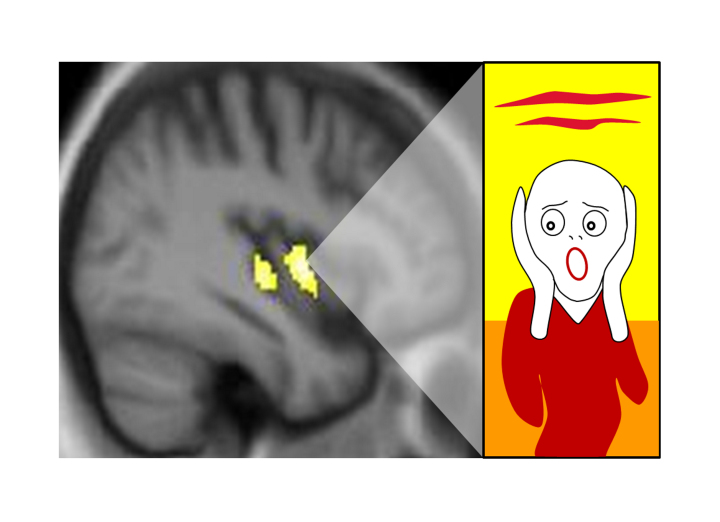
Symptoms suggesting altered pain and temperature processing have been described in dementia diseases. Using a semi-structured caregiver questionnaire and MRI voxel-based morphometry in patients with frontotemporal degeneration or Alzheimer’s disease, Fletcher *et al.* show that these symptoms are underpinned by atrophy in a distributed thalamo-temporo-insular network implicated in somatosensory processing.

## Introduction

The frontotemporal lobar degenerations (FTLDs) are a diverse group of proteinopathies characterized by selective degeneration of distributed brain networks involving the frontal and temporal lobes. Altered processing of sensory signals is an important feature of these diseases (Bathgate *et al.*, 2001; Snowden *et al.*, 2001; Pijnenburg *et al.*, 2004; Jesso *et al.*, 2011; Omar *et al.*, 2011*a*, 2013; Rohrer *et al.*, 2012; Fletcher *et al.*, 2013; Downey *et al.*, 2014; Landqvist Waldo *et al.*, 2014; Perry *et al.*, 2014; Woolley *et al.*, 2014; Zhou and Seeley, 2014). Patients commonly fail to interpret emotional and social cues correctly (Jesso *et al.*, 2011; Omar *et al.*, 2011*a*; Kumfor and Piguet, 2012; Zhou and Seeley, 2014) and may show obsessional attachment to particular stimuli such as sweet foods (Woolley *et al.*, 2014) or music (Fletcher *et al.*, 2013) suggesting a generic disturbance in processing reward and attributing hedonic valence (Perry *et al.*, 2014). However, several series have documented symptoms that might signify a more fundamental abnormality in coding somatosensory signals, in particular pain and temperature, by patients with FTLD (Bathgate *et al.*, 2001; Snowden *et al.*, 2001; Ahmed *et al.*, 2015). Unpleasant somatic symptoms, often with a nociceptive component (including non-specific unexplained headaches, musculoskeletal, abdominal or urogenital discomfort, vague migrating pains, chest pain and pruritus), have been described in a substantial proportion of patients with FTLD and may be early and prominent (Snowden *et al.*, 2001); such symptoms may be particularly salient in the syndromes of semantic dementia and behavioural variant frontotemporal dementia (FTD), especially with *C9orf72* mutations (Landqvist Waldo *et al.*, 2013). Patients may exhibit strikingly abnormal ‘sensory behaviours’, especially reduced responsiveness to painful stimuli in behavioural variant FTD and exaggerated responses to pain in semantic dementia (Bathgate *et al.*, 2001; Snowden *et al.*, 2001). Symptoms suggesting disturbed thermoregulation have also been reported with high prevalence in behavioural variant FTD and semantic dementia (Ahmed *et al.*, 2015). Limited psychophysical evidence has suggested overall increases in pain threshold and tolerance in FTD, albeit with considerable individual variation (Carlino *et al.*, 2010).

Such reports suggest that pain and temperature responsiveness are commonly though variably altered in FTLD and are of considerable interest on both neurobiological and clinical grounds. The neuroanatomical correlates of pain processing in the healthy brain comprise a distributed network with critical hubs in thalamus and insula and somatosensory, prefrontal, anterior temporal, limbic and subcortical connections (Peyron *et al.*, 2000; Craig, 2002; Herde *et al.*, 2007; Moulton *et al.*, 2012). Temperature sensibility is mediated by a closely overlapping network (Craig, 2002; Moulton *et al.*, 2012). Together these networks have a core role in regulation of bodily homeostasis: current neurobiological formulations emphasize convergent processing of somatic and visceral pain and thermoregulatory signals as functionally interdependent aspects of interoception (Craig, 2002, 2009). Separable network components underpin sensory gating and representation (thalamus, posterior insula, somatosensory cortex), arousal and attention (thalamus, anterior cingulate), evaluative and contextual processing (anterior insula, anterior cingulate, anterior temporal cortex, amygdala, hippocampus), and programming behavioural responses (anterior cingulate, orbitofrontal and prefrontal cortices) (Greenspan and Winfield, 1992; Peyron *et al.*, 2000; Singer *et al.*, 2004; Brooks *et al.*, 2005; Höistad and Barbas, 2008; Craig, 2009; Mazzola *et al.*, 2009, 2012; Isnard *et al.*, 2011; Moulton *et al.*, 2012; Meerwijk *et al.*, 2013). These networks overlap extensively with networks targeted by the pathological process in FTLD. Impaired body schema integrity has recently been demonstrated in patients with *C9orf72* mutations (Downey *et al.*, 2012, 2014) and may be a generic pathophysiological mechanism of somatic delusions, somatization and other neuropsychiatric symptoms in these patients, due to disruption of a core thalamo-cortico-cerebellar network (Mahoney *et al.*, 2012; Lee *et al.*, 2014); involvement of thalamus, in particular, has emerged as a consistent and early signature of this mutation subgroup (Rohrer *et al.*, 2015). Impaired appraisal of salient sensory objects and events is integral to the clinical syndromes of semantic dementia and behavioural variant FTD, notably in association with right anterior temporal lobe atrophy (Bathgate *et al.*, 2001; Snowden *et al.*, 2001; Chan *et al.*, 2009). Considered together, this evidence suggests that pain and temperature pathophysiology may link brain network disintegration with clinical symptoms in these diseases. However, in contrast with the well-characterized central pain syndromes attending focal lesions of thalamocortical circuitry (Schmahmann and Leifer, 1992; Blomqvist *et al.*, 2000; Borsook, 2012), the phenomenology of pain and temperature processing and their neuroanatomical bases have not been studied in detail in FTLD. Moreover, altered experience of pain and temperature might be predicted in other neurodegenerative diseases that disrupt the integrity of distributed networks that process pain and temperature (Borsook, 2012). A notable test case is Alzheimer’s disease. Limited available information suggests that sensory encoding and perception of pain are retained in Alzheimer’s disease, at least in early to moderate stage disease, with engagement of a similar central nociceptive network to healthy older individuals (Cole *et al.*, 2006); however, patients’ pain tolerance has been variously reported as unaltered, increased or diminished (Cole *et al.*, 2006, 2011; Borsook, 2012; Jensen-Dahm *et al.*, 2014) and semantic processing of pain concepts may also be diminished (Oosterman *et al.*, 2014).

Here we addressed these issues in cohorts of patients representing the major syndromes of FTLD and typical Alzheimer’s disease. Symptoms suggesting altered pain and temperature processing were characterized using a semi-structured pro forma administered to patients’ caregivers. Structural neuroanatomical correlates of these symptoms were assessed using voxel-based morphometry (VBM) of patients’ brain magnetic resonance images. We hypothesized that pain and temperature symptoms would be over-represented in behavioural variant FTD and semantic dementia versus progressive non-fluent aphasia (PNFA) and Alzheimer’s disease, and more specifically, in patients with *C9orf72* mutations versus other disease groups; and that behavioural variant FTD and semantic dementia have overlapping but differentiable symptom profiles characterized by blunted versus heightened pain and temperature responsiveness, respectively. We further hypothesized that pain and temperature symptoms in the FTLD cohort and in Alzheimer’s disease would be associated with grey matter atrophy in the distributed network previously implicated in pain and temperature processing in the healthy brain. More specifically, we hypothesized an association of insular atrophy with symptoms across syndromes (Peyron *et al.*, 2000; Craig, 2002; Zhou and Seeley, 2014); and partly separable neuroanatomical associations, targeting thalamus in *C9orf72* mutations, more anterior cortical regions in other FTLD subgroups and more posterior somatosensory cortical association areas in Alzheimer’s disease (Cole *et al.*, 2006; Chan *et al.*, 2009; Downey *et al.*, 2014; Lee *et al.*, 2014).

## Materials and methods

### Patient characteristics

Fifty-eight patients with FTLD (25 female, aged 52–84 years) and 20 patients with Alzheimer’s disease (eight female, aged 53–74 years) were assessed consecutively over a 3-year interval via a tertiary Cognitive Disorders Clinic. All fulfilled consensus diagnostic criteria for a syndrome of FTLD (Gorno-Tempini *et al.*, 2011; Rascovsky *et al.*, 2011) (behavioural variant FTD, *n = *21; semantic dementia, *n = *17; PNFA, *n = *20) or for Alzheimer’s disease led by decline in episodic memory (Dubois *et al.*, 2007). The syndromic diagnosis was supported in each case by detailed clinical and neuropsychological evaluation following a standard protocol referenced to a historical, age-matched healthy control group (Table 1) and further corroborated by CSF and brain amyloid PET imaging findings (ratio of total tau: amyloid-β_1-42_ levels >1 in 14/14 Alzheimer’s disease cases and <0.8 in 13/13 FTLD cases; florbetapir PET-negative for amyloid deposition in 7/7 FTLD cases for which data were available). All patients had MRI profiles of regional brain atrophy concordant with their clinical diagnosis; no patient had radiological evidence of significant or strategic vascular damage. Genetic screening revealed 11 patients with a pathogenic mutation (six *C9orf72*; five *MAPT*). All patients with a genetic mutation presented with behavioural variant FTD apart from one patient with a *C9orf72* expansion who presented with PNFA.

**Table 1:** General demographic and neuropsychological data for patient subgroups

Characteristic	FTLD: pain / temperature		AD: pain / temperature		Healthy controls^a^
	Symptoms	No symptoms	Symptoms	No symptoms	
**General demographics**					
*n*: total (F:M)	31(10:21)^b^	27 (15:12)	9 (2:7)^c^	11 (6:5)	50 (23:27)
*n*: syndromes bvFTD / SD / PNFA	15/ 11 / 5	6 / 6 / 15	NA	NA	NA
*n*: no mutation / *C9orf72 / MAPT*	24 / 6 / 2	23 / 0 / 3	NA	NA	NA
Age (years)	65.4 (52–84)	64.8 (47–80)	63.8 (53–71)	65 (57–74)	67.5 (54–80)
Education (years)	13.9 (11–20)	15.2 (11–21)	13 (11–17)	15 (12–17)	15.2 (10–18)
Symptom duration (years)	6.5 (3–21)	4.8 (2–18)	5 (2–8)	5.5 (4–9)	NA
MMSE	**21.1 (4–30)**	**21.9 (1–30)**	**20 (13–25)**	**22.5 (14–29)**	29.6 (28–30)
**MRI profiles****^d^**					
Temporal lobe atrophy (L:R:symm)	22(8:5:9)	16(12:1:3)	6(0:0:6)	9(0:0:9)	NA
Frontal lobe atrophy (L:R:symm)	10(3:2:5)	11(6:1:4)	0	0	NA
Parietal lobe atrophy (L:R:symm)	3(1:0:2)	0	2(0:0:2)	1(0:0:1)	NA
**General intellect**					
Verbal IQ	**76 (40–126)**	**78 (55–119)**	**86 (55–115)**	**93 (55–120)**	120 (101–137)
Performance IQ	**91 (65–136)**	**101 (69–134)**	**81 (59–125)**	**92 (63–119)**	115 (84–141)
**Episodic memory**					
RMT words (/50)	**34 (20–49)**	**37 (18–47)**	**29 (17–42)**	**32 (24–50)**	48 (39–50)
RMT faces (/50)	31 (24–50)*	**36 (25–47)**	**32 (18–45)**	**39 (24–46)**	43 (30–50)
**Executive function**					
Stroop word (90 s)	**35 (16–90)**	**39 (18–90)**	**43 (17–79)**	**36.6 (17–58)**	22.7 (15–53)
Stroop inhibition (180 s)	**100 (48–180)**	**105 (48–180)**	**143 (42–180)**	**101.4 (30–180)**	57.6 (35–103)
Digit span reverse (/12)	**3.7 (0–7)**	**4 (0–7)**	**3 (1–6)**	**3.6 (1–7)**	5 (3–7)
**Semantic processing**					
BPVS (/150)	**98 (2–149)**	**118 (8–149)**	126 (76–146)	132 (52–147)	147 (137–150)
Synonyms (/50)	**34 (12–50)****^b^**	**38 (20–49)**	**41 (30–49)**	**47.5 (46–49)**	48 (36–50)
**Visuospatial**					
VOSP object decision (/20)	16 (8–20)	17 (10–20)	**16 (10–18)**	**15 (7–19)**	18 (12–20)

Mean (range) data are shown unless otherwise indicated and maximum scores on neuropsychology tests are also indicated in parentheses. Significant differences (*P* < 0.05) between patients and controls are in bold.

^a^Historical age-matched group.

^b^Five patients with altered pain responses only, 13 with altered temperature responses only, 13 with both (see Table 2).

^c^Six patients with altered temperature responses only, three with alteration of both pain and temperature responses.

^d^Blinded visual rating of brain MRI scans (L:R:symm, number of cases with relatively focal lobar atrophy predominantly left-sided, right-sided or relatively symmetric; note lobar involvement not mutually exclusive).

*Significantly (*P* < 0.05) different from non-symptomatic patients with FTLD.

AD = syndrome of Alzheimer’s disease led by decline in episodic memory; BPVS = British Picture Vocabulary Scale; bvFTD = behavioural variant FTD; F = female; M = male; MMSE = Mini-Mental State Examination score; NA = not applicable; RMT = Recognition Memory Test; SD = semantic dementia; temp = temperature; VOSP = Visual Object and Space Perception battery.

Patients’ caregivers completed a semi-structured questionnaire designed to identify symptoms suggesting altered pain or temperature processing (altered experience of pain or temperature) developing since the onset of their illness (Supplementary Table 1). This questionnaire recorded caregiver descriptions of patients’ symptoms and initially sought to capture any unexplained unpleasant physical symptoms more generally, before focusing explicitly on altered behavioural responses to pain or temperature variations. Questionnaire data were analysed off-line to determine the nature of any alteration in pain or temperature responsiveness and its directionality (increased versus decreased), based on caregiver descriptions of patients’ overt verbal and non-verbal behaviours. Chi-square tests were used to compare categorical differences in symptom prevalence and linear regression was used to compare differences in background demographic and neuropsychological measures between groups. In addition, we assessed for any correlation between pain and temperature symptoms and any alteration in hedonic processing in the domains of music and environmental sounds, as previously recorded for this patient cohort (Fletcher *et al.*, 2015).

All participants gave informed consent to be involved in the study, which was approved by the local institutional ethics committee in accordance with the Declaration of Helsinki.

### Brain MRI acquisition and analyses

At the time of questionnaire data collection each patient underwent volumetric brain MRI on a 3.0 T Siemens scanner using a 32-channel phased-array head coil. A sagittal 3D magnetization prepared rapid gradient echo T_1_-weighted volumetric MRI (echo time/repetition time/inversion time 2.9/2200/900 ms, dimensions 256 × 256 × 208, voxel size 1.1 × 1.1 × 1.1 mm) was acquired. In all cases, volumetric scans were assessed visually in all planes to ensure adequate coverage and to exclude artefacts or significant motion.

To assess any relation between individual brain atrophy profile and development of pain and temperature symptoms, each patient’s brain magnetic resonance scan was reviewed by two experienced cognitive neurologists (P.D.F., J.D.W.) while blinded to symptomatic and clinical syndromic status. In each case, the presence of relatively focal brain atrophy (disproportionate to more diffuse background atrophy) and the direction of any cerebral hemispheric asymmetry on visual inspection were recorded for the frontal, temporal and parietal lobes. Any apparent right:left directionality of atrophy was assessed for each lobar region using chi-square tests.

Preprocessing of patients’ brain magnetic resonance images for VBM was performed using New Segment (Ashburner and Friston, 2005) and the DARTEL (Ashburner, 2007) toolbox of SPM8 (www.fil.ion.ucl.ac.uk/spm) running under Matlab7.0®. Segmentation, normalization and modulation of grey and white matter images were performed using default parameter settings. Images were smoothed using a Gaussian full-width at half-maximum of 6 mm. To adjust for individual differences in global grey matter volume during subsequent analysis, total intracranial volume was calculated for each participant by summing grey matter, white matter and CSF volumes following segmentation of all three tissue classes. A study-specific group mean template brain image was created by warping all native space whole-brain images to the final DARTEL template and calculating the average of the warped brain images.

Voxel intensity (grey matter volume) was modelled for the combined FTLD cohort and for the Alzheimer’s disease cohort: firstly, as a function of presence versus absence of any symptoms suggesting altered pain or temperature processing; and as a function of presence versus absence of symptoms for pain and for temperature separately. Participant age, total intracranial volume, Mini-Mental State Examination score (as a global measure of disease severity) and syndromic group membership (where relevant) were included as covariates of no interest in the models. In addition, in light of recent evidence suggesting a distinct pathophysiological signature of *C9orf72*-associated FTLD (Downey *et al.*, 2014; Lee *et al.*, 2014), we performed a subanalysis of the symptomatic FTLD cohort contrasting patients with and without *C9orf72* mutations. To help protect against voxel drop-out due to potentially marked local regional atrophy, a customized explicit brain mask was applied based on a specified ‘consensus’ voxel threshold intensity criterion (Ridgway *et al.*, 2009) whereby a voxel was included in the analysis if grey matter intensity at that voxel was >0.1 in >70% of participants (rather than in all participants, as with the default SPM8 mask).

Statistical parametric maps of regional grey matter volume correlating with pain and temperature symptoms were assessed using two prescribed criteria, each thresholded at *P < *0.05 after family-wise error (FWE) correction for multiple voxel-wise comparisons. Maps were first assessed after correction over the whole brain volume, to determine any associations that emerged without taking the evidence of previous studies into account. Maps were next assessed after correction within a regional small volume of interest, taking account of previous evidence and our specific anatomical hypotheses: this single anatomical small volume combined structures in both cerebral hemispheres consistently identified as critical for interoceptive and homeostatic processing of pain and temperature in the healthy brain, namely, insular cortex and thalamus (Lenz *et al.*, 1993; Davis *et al.*, 1999; Craig *et al.*, 2000; Brooks *et al.*, 2005; Kim *et al.*, 2007; Isnard *et al.*, 2011; Mazzola *et al.*, 2012). Relevant anatomical subregions were customized from the Oxford/Harvard brain maps in FSLview v3.1 (Desikan *et al.*, 2006; Jenkinson *et al.*, 2012) to fit the group mean template brain image. The overall distribution of grey matter atrophy in key disease subgroups with and without pain and temperature symptoms was assessed relative to healthy controls in a separate VBM analysis (further details in the online Supplementary material).

## Results

### Analysis of pain and temperature symptoms

Characteristics of the patient cohort are summarized in Table 1 and a detailed analysis of symptoms is presented in Table 2; extracts from caregiver questionnaire reports for individual patients are presented in Supplementary Table 2.

**Table 2:** Detailed description of the symptomatic patient cohort

Syndromic diagnosis	*n*	MRI profile: focal atrophy^a^			Symptom category	Response shift
		TL	FL	PL		
		L/R/symm	L/R/symm	L/R/symm	P/T/both	inc/dec/both^b^
Behavioural variant FTD	15	1/2/5	2/2/3	1/0/0	4/7/4	6/6/3
Semantic dementia	11	6/2/3	0	0/0/1	1/3/7	8/1/2
PNFA	5	1/1/1	1/0/2	0/0/1	0/3/2	3/0/2
Alzheimer’s disease	9	0/0/6	0	0/0/2	0/6/3	7/0/2

*Blinded visual rating of brain MRI scans (L:R:symm, number of cases with relatively focal lobar atrophy predominantly left-sided, right-sided or relatively symmetric; note lobar involvement not mutually exclusive).

^b^Variably increased or decreased responsiveness within or between modalities.

dec = decreased; FL = frontal lobe atrophy; inc = increased; L = left; N = normal; P = symptoms of altered pain experience; PL = parietal lobe atrophy; R = right; symm = relatively symmetric; T = symptoms of altered temperature experience; TL = temporal lobe atrophy.

Symptoms suggesting abnormalities of pain and/or temperature processing were reported in 31/58 patients with FTLD (53% of the whole FTLD cohort) and in 9/20 patients with Alzheimer’s disease (45% of the Alzheimer’s disease cohort). In both FTLD and Alzheimer’s disease cohorts, altered responses to temperature variations were more frequently reported than altered responses to pain. While patients with FTLD (13/31 cases, 41%) and Alzheimer’s disease (3/9, 33%) commonly had altered responses both to pain and temperature, only patients with FTLD (5/31 cases, 16%) had altered pain responses alone. Within the FTLD cohort, symptoms suggesting altered pain or temperature processing were statistically significantly more common in the behavioural variant FTD group (15/21 cases, 71%) and semantic dementia group (11/17 cases, 65%) than in the PNFA group (5/20 cases, 25%: *P < *0.05 all comparisons): accordingly, behavioural variant FTD and semantic dementia phenotypes were relatively over-represented in the symptomatic FTLD subgroup (Table 1). Of the genetic FTLD subgroups, patients with *C9orf72* mutations were over-represented in the symptomatic subgroup, reporting symptoms suggesting altered pain or temperature processing in all (6/6) cases; whereas these symptoms were recorded less frequently (2/5 cases) for patients with *MAPT* mutations.

Caregiver reports (Supplementary Table 2) revealed a diverse phenomenology of altered pain and temperature experience among patients in the symptomatic cohort. Pain symptoms were variably reported as arising from the external environment or from within the patient’s own body; while temperature symptoms were mainly described as subjective discomfort relative to the ambient environment and only occasionally referred to thermal touch *per se*. Symptoms varied widely in intensity and frequency. Both increased responsiveness and decreased responsiveness to pain and temperature variations were described, as well as responses that were variably increased or decreased within or between modalities. Within the temperature modality, patients more often developed a dislike of cold (rather than warm) environments. The directional preponderance of altered pain and temperature responsiveness varied between syndromic groups: within the behavioural variant FTD group, decreased responsiveness and increased responsiveness to pain and temperature variations were equally frequent (each reported in six cases, 40%); whereas increased responsiveness was more commonly described within the semantic dementia group (8/11 cases, 73%), the PNFA group (3/5 cases, 60%) and the Alzheimer’s disease group (7/9 cases, 78%; see Table 2). More complex bidirectional shifts in pain and temperature responses were also described in all syndromic groups. The small *C9orf72* mutation subgroup described symptoms similar to those reported for the cohort as a whole (Supplementary Table 2).

When neuropsychological profiles in the patient subgroups within the FTLD and Alzheimer’s disease cohorts were compared according to the presence or absence of pain and temperature symptoms, the symptomatic FTLD group showed significantly (*P < *0.01) greater impairment of face memory than the non-symptomatic FTLD subgroup; the subgroups within each disease cohort were otherwise similar overall (Table 1). Thyroid function (available for 66/78 patients including 34/40 patients with pain and temperature symptoms) was normal in all cases assessed. Three patients in the FTLD cohort with pain and temperature symptoms underwent nerve conduction studies, which were normal in all cases. The presence of pain and/or temperature symptoms was positively correlated with altered liking for music (*P = *0.03) but not environmental sounds (*P = *0.8).

### Neuroanatomical correlates of altered pain and temperature processing

Visual review of individual patient MRI scans (summarized in Tables 1 and 2) revealed an over-representation of cases with relatively focal temporal lobe atrophy in the subgroup of patients with FTLD and pain and temperature symptoms in comparison to the group without such symptoms. In particular, right-sided temporal lobe atrophy was more common in the subgroup of patients with pain and temperature symptoms than the subgroup without symptoms (case ratio 5:1; see Table 1). However, this apparent disproportion was not statistically significant (*P* > 0.05). Focal temporal lobe atrophy was frequent in all three FTLD syndromes within the symptomatic cohort but concentrated (as anticipated) in the semantic dementia subgroup. These findings were further corroborated in the VBM analysis mapping overall grey matter atrophy profiles in FTLD subgroups with and without pain and temperature symptoms versus healthy controls (Supplementary Fig. 1 and Supplementary Table 3). Disproportionate temporal lobe atrophy was also frequent in patients with Alzheimer’s disease and pain and temperature symptoms; however, in contrast to the FTLD cases, none of these Alzheimer’s disease patients exhibited asymmetric temporal lobe involvement nor was there any temporal lobe predilection for symptomatic versus non-symptomatic Alzheimer’s disease cases.

**Figure 1:**
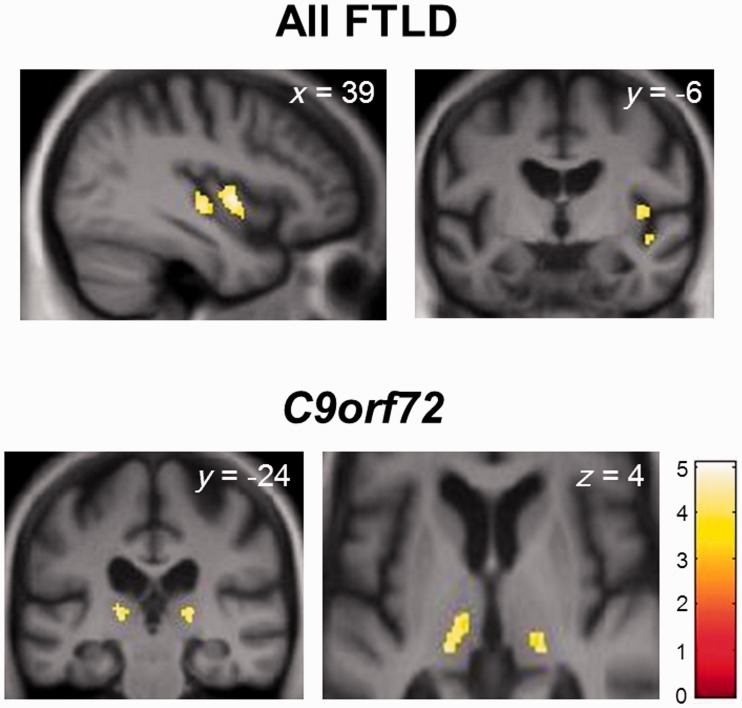
**Statistical parametric maps (SPMs) showing regional grey matter atrophy significantly associated with altered pain and/or temperature responsiveness in the FTLD cohort.** See also Table 3. SPMs are based on the contrast between patient subgroups with and without symptoms in the combined cohort (*All FTLD, top*) and in patients with *C9orf72* mutations (*C9orf72*; all symptomatic) versus symptomatic patients without *C9orf72* mutations (*bottom*). SPMs are thresholded for display purposes at *P < *0.001 uncorrected for multiple comparisons over the whole brain and rendered on sections of a group mean T_1_-weighted magnetic resonance brain template image in MNI standard space; coordinates (mm) of the plane of each section are indicated and the right hemisphere is shown on the right of the coronal sections and the axial section (*bottom right*, magnified to show thalamic anatomy). *Z*-scores for the SPMs are scaled according to the colour bar.

Regional grey matter correlates of pain and temperature symptoms from the VBM analysis are summarized in Table 3 and statistical parametric maps are shown in Fig. 1. No grey matter correlates of pain and temperature symptoms were identified at the prescribed corrected significance threshold (*P < *0.05_FWE_) at the level of the whole brain. However, within the combined FTLD cohort, the presence of any alteration in pain or temperature responsiveness was significantly associated with atrophy of right mid insula (and borderline significant also for right posterior insula) when thresholded at *P < *0.05_FWE_ after correction within the prespecified anatomical region of interest; no significant grey matter associations were identified for pain symptoms or for temperature symptoms separately, at the prescribed threshold. In the separate VBM subanalysis of patients with *C9orf72* expansions contrasted with other FTLD patients showing altered pain or temperature responses (Table 3 and Fig. 1), symptoms due to *C9orf72* mutations were significantly associated with atrophy of right posterior thalamus (and borderline significant also for left posterior thalamus; *P < *0.05_FWE_ within the prespecified anatomical region of interest). No other grey matter associations were identified at the prescribed significance threshold.

**Table 3:** Voxel-based morphometric correlates of altered pain and temperature processing in FTLD

Grey matter association	Brain region	Side	Cluster (voxels)	Peak (mm)			*Z*-score	*P*-value
				***x***	***y***	***z***		
All FTLD	Mid insula	R	227	40	−1	0	4.37	0.02
	Posterior insula	R	105	39	−18	−2	4.05	0.055
*C9orf72* mutations	Posterior thalamus	R	66	20	−24	3	3.73	0.03
		L	115	−18	−25	1	3.55	0.055

Significant regional correlates of altered pain and temperature processing (grey matter atrophy associated with any symptoms suggesting altered responsiveness to pain and/or temperature) are based on contrasts over the whole frontotemporal lobar degeneration (FTLD) cohort (all symptomatic versus all asymptomatic patients) and in patients with *C9orf72* mutations (all symptomatic versus symptomatic patients without *C9orf72* mutations). Associations are reported after correction for multiple voxel-wise comparisons within the prespecified anatomical small volume of interest; all significant clusters >40 voxels are shown and peak (local maximum) coordinates are in MNI standard stereotactic space (see also Fig. 1 and further details in Supplementary Fig. 2).

To identify any less robust but potentially relevant neuroanatomical associations of pain and temperature symptoms in the patient cohort, we performed a separate *post hoc* exploratory analysis of the VBM data thresholded more leniently at *P < *0.001 uncorrected for multiple voxel-wise comparisons over the whole brain. At this more lenient threshold, additional regional grey matter correlates were observed (Supplementary Fig. 2 and 3). Pain and temperature symptoms in the combined FTLD cohort and in the subgroup not associated with *C9orf72* mutations were additionally associated with atrophy of right anterior temporal cortex; while temperature symptoms (but not pain symptoms) in the FTLD cohort were associated with atrophy of right mid-insula (Supplementary Fig. 2). Pain and temperature symptoms within the smaller Alzheimer’s disease cohort were associated with grey matter atrophy in left angular gyrus (Supplementary Fig. 3) using this relaxed criterion.

## Discussion

Here we have shown that altered experience of pain and temperature is common in the major dementia syndromes. Altered behavioural responses to both pain and temperature were frequently reported but more often reported for temperature than pain. In line with our prior hypotheses and previous work (Bathgate *et al.*, 2001; Snowden *et al.*, 2001, 2013; Pijnenburg *et al.*, 2004; Mahoney *et al.*, 2012; Ahmed *et al.*, 2015; Downey *et al.*, 2014; Landqvist Waldo *et al.*, 2014), certain syndromic signatures were identified. Pain and temperature symptoms were commonly described across FTLD syndromes, exhibited by the majority of patients with behavioural variant FTD and semantic dementia and most frequently in the molecular subtype represented by *C9orf72* mutations. Patients with Alzheimer’s disease exhibited similar symptoms in a somewhat lower proportion (45%) of cases. The directionality of symptoms (heightened versus diminished responses to pain and temperature) varied across the cohort but was also modulated by syndrome: increased responsiveness to pain or temperature variations was most often reported in the semantic dementia, PNFA and Alzheimer’s disease groups whereas decreased responsiveness was most often associated with behavioural variant FTD, again consistent with previous evidence (Bathgate *et al.*, 2001; Snowden *et al.*, 2001; Carlino *et al.*, 2010). Occurrence of symptoms was not simply attributable to disease severity or other demographic or general neuropsychological factors. Though not quantified here, the reported intensity and frequency of symptoms varied widely among individuals in each syndromic group.

Pain and temperature symptoms were frequently accompanied by focal (and particularly, right-sided) temporal lobe atrophy in individual patients with FTLD, as anticipated from previous work and consistent with the more severe face processing deficit in this FTLD subgroup (Snowden *et al.*, 2001; Chan *et al.*, 2009; Omar *et al.*, 2011*b*). However, asymmetric temporal lobe atrophy was not a *sine qua non* for development of such symptoms, as illustrated by the Alzheimer’s disease cases. In line with these findings and with previous studies in the healthy brain (Peyron *et al.*, 2000; Craig, 2002; Singer *et al.*, 2004; Henderson *et al.*, 2007; Herde *et al.*, 2007; Isnard *et al.*, 2011; Borsook, 2012; Moulton *et al.*, 2012), a VBM group analysis of patients’ brain MRIs delineated a distributed network of brain regions where atrophy was associated with altered responsiveness to pain or temperature. The most robust network correlates of pain and temperature symptoms comprised right-lateralized grey matter areas in mid and posterior insula in the combined FTLD group and bilateral posterior thalamus in the *C9orf72* mutation group.

These findings substantiate current formulations of the neural organization of central somatosensory and homeostatic signal processing (Craig, 2002, 2009; Borsook, 2012; Preusser *et al.*, 2015). Peripheral somatic and visceral sensory afferents conveying pain and thermal information relay via postero-lateral thalamic nuclei to somatosensory cortex (Brodmann area 3a) and dorsal posterior insula (Craig, 2002). While primary somatosensory cortex may process tactile events (Preusser *et al.*, 2015), classical models emphasizing somatosensory labelled lines have been incorporated by current models that emphasize the intimate association of pain and thermal information and their integration as joint aspects of interoception, salient sensory phenomena that are potentially critical for signalling body homeostasis (Craig, 2002, 2009). The posterior insula is a central network hub for integration of these homeostatic signals to map an interoceptive ‘image’ of body state (Craig, 2002). Focal lesions of posterior thalamus and posterior insula are well known to produce central pain syndromes and microstimulation of these regions may produce pain and thermal sensations (Blomqvist *et al.*, 2000; Mazzola *et al.*, 2009, 2012; Sprenger *et al.*, 2012). The thalamo-insular network plays a broader role in integrating external and interoceptive sensory signals to generate a coherent body schema that defines bodily integrity and agency in relation to the environment (Lenz *et al.*, 1993; Davis *et al.*, 1999; Banzett *et al.*, 2000; Craig *et al.*, 2000, 2002, 2009; Critchley *et al.*, 2000, 2011; Brooks *et al.*, 2005; Critchley, 2005; Henderson *et al.*, 2007; Seeley *et al.*, 2007*a*, *b*, 2009; Corbetta *et al.*, 2008; Sridharan *et al.*, 2008; Bjornsdotter *et al.*, 2009; Mazzola *et al.*, 2009, 2012; Menon and Uddin, 2010; Beissner *et al.*, 2013).

Both insula and thalamus are involved by the pathological process in FTLD (Chow *et al.*, 2008; Zhou *et al.*, 2010; Garibotto *et al.*, 2011). In this regard, it is particularly pertinent that posterior thalamic atrophy emerged here as a signature of pain and temperature symptoms with *C9orf72* mutations. While the clinical and neuroanatomical phenotypic spectrum of *C9orf72*-associated disease is protean, neuropsychiatric disturbances are often early and prominent and may be underpinned by disintegration of a large-scale cortico-thalamo-cerebellar brain network (Downey *et al.*, 2012; Mahoney *et al.*, 2012; Snowden *et al.*, 2012, 2013; Takada and Sha, 2012; Whitwell *et al.*, 2012; Lee *et al.*, 2014; Rohrer *et al.*, 2015). Deranged body schema processing is a candidate pathophysiological mechanism linking network pathology with clinical features in *C9orf72* mutations (Downey *et al.*, 2014), and disturbed pain processing due to thalamic dysfunction might be a critical signal of this more general impairment in representing bodily integrity. It would be intriguing if this clinical phenotype had a specific micro-anatomical marker (Blomqvist *et al.*, 2000). Pathological data have demonstrated heavy thalamic involvement in a series of *C9orf72* mutation cases reporting a high frequency of unexplained somatic and visceral pains during life (Landqvist Waldo *et al.*, 2013). Although precise localization was not possible in this study, the posterior thalamic correlate identified in the *C9orf72* mutation group here closely approximates several potentially relevant thalamic subregions. These include pulvinar, which may regulate cortical ‘set’ for interpreting painful stimuli (Shipp, 2003); and the posterior portion of the ventral medial thalamic nucleus, which is likely to serve as a dedicated spinothalamocortical relay for pain and temperature sensations and may play a fundamental role in signalling physiological body states (Blomqvist *et al.*, 2000).

Caution is needed in interpreting the additional neuroanatomical correlates identified in this study, as these were exploratory and substantially less statistically robust. However, involvement of right anterior temporal cortex in the FTLD group (and more particularly, the subgroup of patients without *C9orf72* mutations) here was corroborated both by inspection of individual magnetic resonance brain images and the relaxed VBM analysis, and chimes with previous evidence implicating this region in non-verbal sensory semantic (including pain and somatic) processing (Chan *et al.*, 2009; Goll *et al.*, 2010; Omar *et al.*, 2010, 2013; Hsieh *et al.*, 2011). Unpleasant somatic and visceral sensory experiences occur with focal damage involving this region (Naga *et al.*, 2004; Erickson *et al.*, 2006), while anterior temporal lobe dysfunction associated with migraine enhances thalamo-cortical connectivity (Moulton *et al.*, 2011). The temporal lobe may play a key role in contextualizing unpleasant sensory experience by linking these to other data on current bodily state, previous autobiographical experiences and stored conceptual (including social normative) knowledge (Rankin *et al.*, 2006; Zahn *et al.*, 2009; Aminoff *et al.*, 2013; Irish *et al.*, 2014) and by engaging a distributed anterior fronto-temporal appraisal network (Guo *et al.*, 2013; Zhou and Seeley, 2014). Degradation of this contextualizing function might preclude programming of a coherent, organized behavioural response to pain and temperature variations. The critical linkage of anterior temporal mechanisms with interoceptive processing networks is likely to be mediated via an integrative hub in mid-insular cortex (Craig, 2009), which emerged as a separate neuroanatomical correlate of altered pain and temperature responsiveness here. This region may be involved in generating subjective psychological states via projections to anterior insula, anterior cingulate, orbitofrontal and prefrontal cortices and in programming coherent autonomic effector responses (Craig, 2002, 2009; Grecucci *et al.*, 2013; Zhou and Seeley, 2014). This in turn suggests a brain substrate for autonomic dysregulation in FTLD (Ahmed *et al.*, 2015).

With a similar caveat regarding interpretation of uncorrected data, VBM analysis of the present Alzheimer’s disease cohort revealed a distinct cortical correlate of pain and temperature symptoms in inferior parietal cortex. This cortical region has been implicated in processing pain and in particular, in reorienting brain activity between resting ‘default mode’ and active attention to salient stimuli (Kucyi *et al.*, 2012; Bray *et al.*, 2015). Moreover, the region is a core target of pathology in Alzheimer’s disease (Warren *et al.*, 2012) and may be involved in a range of behavioural features in this disease that remain incompletely characterized. These Alzheimer’s disease-associated behavioural changes include anxiety and hyper-emotionality, features that also typically develop in chronic pain syndromes (Sturm *et al.*, 2013; Kucyi *et al.*, 2014; Pujol *et al.*, 2014). Aberrant activity of temporo-parietal cortex in Alzheimer’s disease might disrupt processing of interoceptive signals, both by amplifying ruminative awareness of body states via the default mode network and by gating activity in insula and anterior networks that reciprocally interact in evaluating salient stimuli (Grecucci *et al.*, 2013; Letzen *et al.*, 2013; Zhou and Seeley, 2014). The relative prominence of temperature (relative to pain) symptoms in the Alzheimer’s disease cohort here supports this interpretation, as under most circumstances thermal comfort or distress reflects the degree of perceived mismatch between one’s own body temperature and the environment; temperature sensibility might therefore be regarded as a probe of interoceptive signal processing *par excellence* (Craig, 2002).

Our focus on behaviourally relevant symptoms underlines several challenges in studying patients’ experience of pain and temperature. As conceptualized in contemporary neurobiological models (Craig, 2002, 2009) and illustrated by the present behavioural data, these are complex psychological constructs: alteration in a patient’s responsiveness to pain or temperature variations might reflect altered awareness, tolerance, motivation, behavioural organization or some interaction of these, all potentially dissociable processes. Informant-derived data are subject to bias: caregivers may be more likely to report patients’ behaviour or verbal output where these are heightened rather than attenuated, while certain modalities (such as non-painful thermal touch) are intrinsically less accessible to such reporting. In a number of cases, complex or bidirectional shifts in patient behaviour were described; the attempt to assign a direction to behavioural change is particularly problematic in the case of temperature processing, which might, in principle, reflect altered sensitivity to external heat, cold or both relative to own body temperature. Moreover, structural neuroanatomical correlation using VBM is a rather blunt instrument for investigating such complex network-based processes. In addition to any direct association with atrophy profile, relevant disease effects are likely to reside in connectivity alterations among network elements that are not captured on VBM, perhaps accounting for the absence here of disease effects in anterior cingulate or orbitofrontal regions that might have been anticipated *a priori* (Craig, 2002; Zhou and Seeley, 2014). On the other hand, VBM can identify brain substrates that are critical in the generation of symptoms.

**Figure 2:**
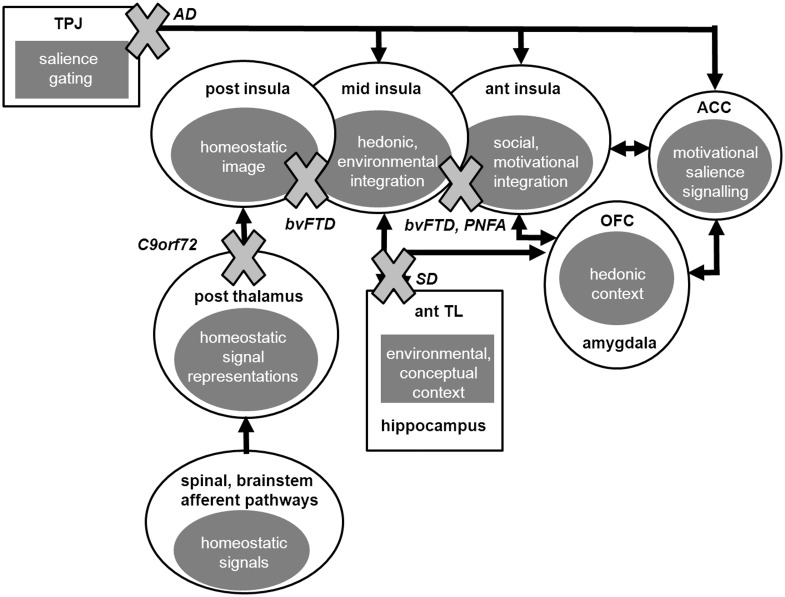
**A schematic synthesis of the effects of dementia syndromes on pain and temperature processing. Based on present data and current formulations of central homeostasis (****Craig, 2002, 2009****; ****Höistad and Barbas, 2008****; ****Borsook, 2012****; ****Zhou and Seeley, 2014)****.** Ellipses indicate core components of the homeostatic processing network, rectangles indicate linked brain regions that modulate processing of homeostatic signals and arrows signify predominant direction of information flow; anatomical regions are labelled alongside their putative roles in the processing hierarchy (grey filled ellipses) and dementia syndromes are labelled (italics) with grey crosses indicating the major locus of dysfunction in that syndrome. According to the proposed synthesis, *C9orf72* mutations target early encoding of pain and temperature signals in thalamo-cortical circuitry; behavioural variant FTD disrupts the relay of body state information from posterior insula and both behavioural variant FTD and PNFA degrade its contextual integration in mid insula and more anterior regions; semantic dementia degrades anterior temporal lobe mechanisms that evaluate stimulus context; and temporo-parietal cortical damage in Alzheimer’s disease leads to abnormally enhanced gating and aberrant salience coding of homeostatic signals. Besides interruption of signalling pathways, degraded (e.g. temporally dysregulated) information flow may also contribute to network dysfunction (Craig, 2009). ACC = anterior cingulate cortex; AD = Alzheimer’s disease; ant = anterior; bvFTD = behavioural variant FTD; OFC = orbitofrontal cortex; post = posterior; SD = semantic dementia; TL = temporal lobe; TPJ = temporo-parietal junction.

Taking the above caveats into account, the present behavioural, MRI and VBM data together allow certain conclusions to be drawn about the breakdown of brain organization for pain and temperature processing in these neurodegenerative diseases. The processing of pain and temperature entails the transformation of sensory data into a complex experiential construct via hierarchical and integrative processing over a series of cortical relays: these stimuli are of fundamental biological significance (demanding high fidelity decoding) and at the same time, richly invested with subjective emotional, mnestic and semantic associations (demanding contextual editing and interpretation). As such, pain and temperature provide a paradigmatic illustration of a key principle of cortical neurobiology (Mesulam, 1998; Craig, 2002, 2009) and an ideal probe of large-scale brain network operations in neurodegenerative disease (Zhou and Seeley, 2014). The present evidence suggests a model for synthesizing neurodegenerative disease effects on these cortical operations that is consistent both with data from normal neurophysiological and functional neuroimaging work and the effects of focal brain lesions (Craig, 2002, 2009; Borsook, 2012). According to this synthesis (Fig. 2), *C9orf72* mutations target early encoding of pain and temperature signals at the level of thalamo-cortical circuitry, accounting for the high proportion of cases reporting relevant symptoms here; while behavioural variant FTD more generally disrupts the relay of body state information from posterior insula and its integration with hedonic and environmental context in mid insula and more anterior regions. This interpretation allows for either abnormally reduced or abnormally increased subjective awareness of homeostatic signals, based on the extent to which network activity is interrupted or continues to transfer noisy signals (a mechanism potentially analogous to pain asymbolia following focal insular lesions: Berthier *et al.*, 1988; Masson *et al.*, 1991). Such noisy processing might involve degraded temporal scheduling of salient sensory and emotional signals, a key function attributed to anterior insula that is vulnerable in FTLD (Wiener and Coslett, 2008; Craig, 2009; Henley *et al.*, 2014). More anterior insular regions are also targeted in PNFA, providing a candidate locus for altered homeostatic awareness in this syndrome (Seeley *et al.*, 2009). Degeneration of anterior temporal lobe mechanisms in semantic dementia impairs contextual processing of minor discomforts via the linkage to mid insula, resulting in aberrant ‘over-valuation’ (decreased tolerance) of such stimuli; while temporo-parietal cortical damage in Alzheimer’s disease leads to aberrant salience coding of homeostatic signals, perhaps via abnormally enhanced gating of interoceptive information between the default mode network and anterior salience network (Zhou and Seeley, 2014).

This model does not exclude the possibility of additional loci (for example, spinal and brainstem pathways) at which neurodegenerative pathologies might degrade pain and temperature signals, with potential consequences for cortical elaboration of these signals (Braak *et al.*, 2007; Olausson *et al.*, 2010). However, the cerebral regions implicated in altered pain and temperature processing here overlap closely with regions previously implicated in social cognition, underlining the close coupling of homeostatic and social signal processing and their joint vulnerability in disease states (Craig, 2009; Grecucci *et al.*, 2013; Zhou and Seeley, 2014). Related phenomena such as social touch (the human analogue of grooming behaviour) are likely to share this brain circuitry and may contribute to deficits of interpersonal awareness in FTLD and other dementias (Craig, 2009). It is increasingly recognized that neurodegenerative diseases disrupt the hedonic valuation of a range of sensory signals. Besides pain and temperature, this spectrum includes signals related to eating and satiety (Whitwell *et al.*, 2007; Woolley *et al.*, 2014), music and other complex sounds (Fletcher *et al.*, 2013, 2015) and somatosensory boundaries (Downey *et al.*, 2012, 2014). It is therefore of interest that homeostatic symptoms in the present patient cohort were correlated with altered hedonic valuation of music. This work has demonstrated neural networks that are engaged jointly by these diverse phenomena and reaffirms the primacy of the thalamo-insular linkage in regulating the interface between homeostatic and environmental contingencies, reward and punishment (Craig, 2002, 2009; Perry *et al.*, 2014; Zhou and Seeley, 2014). Furthermore, pain like other signals in this spectrum is a crucial basis for empathy and understanding of others’ mental states, suggesting a mechanism for co-opting this same brain circuitry to represent selves other than one’s own. Such ‘mentalizing’ activity been proposed as the evolutionary driver for music (Clark *et al.*, 2015) and its disruption is an essential harbinger of many dementias (Rankin *et al.*, 2006; Sturm *et al.*, 2013).

From a clinical perspective, the present work provides a framework for understanding an important category of symptoms that has been under-emphasized in neurodegenerative disease. Our findings underline the prevalence of pain and temperature symptoms across the FTLD spectrum and suggest a syndromic preponderance characterized by blunted versus heightened responsiveness to pain and temperature signals in behavioural variant FTD and semantic dementia, respectively. This syndromic association in turn may suggest a candidate mechanism for the somatization, hypochondriasis and abnormal illness behaviour that these patients frequently exhibit, particularly in the setting of focal right temporal lobe atrophy (Chan *et al.*, 2009): bodily sensations divested of contextual meaning might plausibly drive such behaviours, particularly if compounded by deficits of social comportment (Rankin *et al.*, 2006; Zahn *et al.*, 2009; Irish *et al.*, 2014). Prominent pain and temperature alterations may constitute a clinical signature of *Corf72* mutations. Perhaps more surprisingly, our findings suggest that similar symptoms (particularly affecting thermoregulatory signals) are not uncommon in patients with Alzheimer’s disease and have probably been under-recognized. Aside from any potential value in diagnosing dementia syndromes, improved understanding of patients’ experience of pain and temperature holds clear practical implications for management. The present findings go beyond the simple assumption that cognitive impairment hampers communication of distress—the data suggest that underlying brain mechanisms of pain and temperature processing may be altered in dementia. Patients with dementia may be at increased risk of undetected illness or injury and potentially vulnerable to hypothermia, or other homeostatic derangements. The recognition that homeostatic processing may be significantly degraded in these diseases paves the way for developing strategies to ensure that patients are neither subjected to futile diagnostic procedures nor denied medical attention simply because they are uncomplaining.

This study suggests a number of directions for further work. Future studies should engage larger patient cohorts and assess those cohorts longitudinally, to allow more detailed clinical stratification and validate the diagnostic biomarker potential of pain and temperature alterations; ultimately, histopathological correlation will be required. The Alzheimer’s disease group here was relatively young; further work should extend this population to the wider population of older-onset Alzheimer’s disease. Processing of pain and thermal signals should be directly assessed and clinical rating scales should be verified using quantitative sensory testing and other neurophysiological and autonomic techniques and brain mechanisms should be elucidated using parallel functional neuroimaging paradigms. Rather than relying on a simplified binary classification (symptoms present or absent), future work should code symptom frequency and intensity for parametric correlation with other disease measures. It will also be important to explore patients’ conceptualization of interoceptive signals, as this might yield further signatures of disease [for example, delusional elaboration in association with *C9orf72* mutations (Downey *et al.*, 2014); degraded semantic representations of pain, potentially relevant to a number of diseases (Oosterman *et al.*, 2014)]. Pain and temperature may constitute a useful model system for investigating abnormalities of sensory salience, homeostatic and self schema processing that are core to the pathophysiology of canonical neurodegenerative diseases (Craig, 2002, 2009; Downey *et al.*, 2014; Zhou and Seeley, 2014).

## Supplementary Material

Supplementary Table 1

## Abbreviations

FTD: frontotemporal dementia
FTLD: frontotemporal lobar degeneration
PNFA: progressive non-fluent aphasia
VBM: voxel-based morphometry

## References

[awv276-B1] Ahmed RM Iodice V Daveson N Kiernan MC Piguet O Hodges JR Autonomic dysregulation in frontotemporal dementia. J Neurol Neurosurg Psychiatry 2015; 86: 1048–9. 25550415 10.1136/jnnp-2014-309424

[awv276-B2] Aminoff EM Kveraga K Bar M The role of the parahippocampal cortex in cognition. Trends Cogn Sci 2013; 17: 379–90. 23850264 10.1016/j.tics.2013.06.009 PMC3786097

[awv276-B3] Ashburner J A fast diffeomorphic image registration algorithm. Neuroimage 2007; 38: 95–113. 17761438 10.1016/j.neuroimage.2007.07.007

[awv276-B200] Ashburner J Friston KJ Unified segmentation. Neuroimage 2005; 260: 839–51. 15955494 10.1016/j.neuroimage.2005.02.018

[awv276-B4] Banzett RB Mulnier HE Murphy K Rosen SD Wise RJ Adams L Breathlessness in humans activates insular cortex. Neuroreport 2000; 11: 2117–20. 10923655 10.1097/00001756-200007140-00012

[awv276-B5] Bathgate D Snowden JS Varma A Blackshaw A Neary D Behaviour in frontotemporal dementia, Alzheimer's disease and vascular dementia. Acta Neurol Scand 2001; 103: 367–78. 11421849 10.1034/j.1600-0404.2001.2000236.x

[awv276-B6] Beissner F Meissner K Bar KJ Napadow V The autonomic brain: an activation likelihood estimation meta-analysis for central processing of autonomic function. J Neurosci 2013; 33: 10503–11. 23785162 10.1523/JNEUROSCI.1103-13.2013 PMC3685840

[awv276-B7] Berthier M Starkstein S Leiguarda R Asymbolia for pain: a sensory-limbic disconnection syndrome*.* Ann Neurol 1988; 24: 41–9. 3415199 10.1002/ana.410240109

[awv276-B8] Bjornsdotter M Loken L Olausson H Vallbo A Wessberg J Somatotopic organization of gentle touch processing in the posterior insular cortex. J Neurosci 2009; 29: 9314–20. 19625521 10.1523/JNEUROSCI.0400-09.2009 PMC6665561

[awv276-B9] Blomqvist A Zhang ET Craig AD Cytoarchitectonic and immunohistochemical characterization of a specific pain and temperature relay, the posterior portion of the ventral medial nucleus, in the human thalamus. Brain 2000; 123: 601–19. 10686182 10.1093/brain/123.3.601

[awv276-B10] Borsook D Neurological diseases and pain. Brain 2012; 135: 320–44. 22067541 10.1093/brain/awr271 PMC3281476

[awv276-B11] Braak H Sastre M Bohl JR de Vos RA Del Tredici K Parkinson's disease: lesions in dorsal horn layer I, involvement of parasympathetic and sympathetic pre- and postganglionic neurons. Acta Neuropathol 2007; 113: 421–9. 17294202 10.1007/s00401-007-0193-x

[awv276-B12] Bray S Arnold AE Levy RM Iaria G Spatial and temporal functional connectivity changes between resting and attentive states. Hum Brain Mapp 2015: 36: 549–65. 25271132 10.1002/hbm.22646 PMC6869123

[awv276-B13] Brooks JCW Zambreanu L Godinez A Craig AD Tracey I Somatotopic organisation of the human insula to painful heat studied with high resolution functional imaging. Neuroimage 2005; 27: 201–9. 15921935 10.1016/j.neuroimage.2005.03.041

[awv276-B14] Carlino E Benedetti F Rainero I Asteggiano G Cappa G Tarenzi L Pain perception and tolerance in patients with frontotemporal dementia. Pain 2010; 151: 783–9. 20934811 10.1016/j.pain.2010.09.013

[awv276-B15] Chan D Anderson V Pijnenburg Y Whitwell J Barnes J Scahill R The clinical profile of right temporal lobe atrophy. Brain 2009; 132: 1287–98. 19297506 10.1093/brain/awp037

[awv276-B16] Chow TW Izenberg A Binns MA Freedman M Stuss DT Scott CJ Ramirez J Black SE Magnetic resonance imaging in frontotemporal dementia shows subcortical atrophy. Dem Ger Cogn Disord 2008; 26: 79–88. 10.1159/000144028 18617738

[awv276-B17] Clark CN Downey LE Warren JD Brain disorders and the biological role of music. Soc Cogn Affect Neurosci 2015; 10: 444–52. 24847111 10.1093/scan/nsu079 PMC4350491

[awv276-B18] Cole LJ Farrell MJ Duff EP Barber JB Egan GF Gibson SJ Pain sensitivity and fMRI pain-related brain activity in Alzheimer's disease. Brain 2006; 129: 2957–65. 16951408 10.1093/brain/awl228

[awv276-B19] Cole LJ Gavrilescu M Johnston LA Gibson SJ Farrell MJ Egan GF The impact of Alzheimer's disease on the functional connectivity between brain regions underlying pain perception. Eur J Pain 2011; 15: 568 e1–11. 21257326 10.1016/j.ejpain.2010.10.010

[awv276-B20] Corbetta M Patel G Shulman GL The reorienting system of the human brain: from environment to theory of mind. Neuron 2008; 58: 306–24. 18466742 10.1016/j.neuron.2008.04.017 PMC2441869

[awv276-B21] Craig AD How do you feel? Interoception: the sense of the physiological condition of the body. Nat Rev Neurosci 2002; 3: 655–66. 12154366 10.1038/nrn894

[awv276-B22] Craig AD How do you feel–now? The anterior insula and human awareness. Nat Rev Neurosci 2009; 10: 59–70. 19096369 10.1038/nrn2555

[awv276-B23] Craig AD Chen K Bandy D Reiman EM Thermosensory activation of insular cortex. Nat Neurosci 2000; 3: 184–90. 10649575 10.1038/72131

[awv276-B24] Critchley HD Neural mechanisms of autonomic, affective, and cognitive integration. J Comp Neurol 2005; 493: 154–66. 16254997 10.1002/cne.20749

[awv276-B25] Critchley HD Corfield DR Chandler MP Mathias CJ Dolan RJ Cerebral correlates of autonomic cardiovascular arousal: a functional neuroimaging investigation in humans. J Physiol 2000; 523 (Pt 1), 259–70. 10673560 10.1111/j.1469-7793.2000.t01-1-00259.x PMC2269796

[awv276-B26] Critchley HD Nagai Y Gray MA Mathias CJ Dissecting axes of autonomic control in humans: insights from neuroimaging. Auton Neurosci 2011; 161: 34–42. 20926356 10.1016/j.autneu.2010.09.005

[awv276-B27] Davis KD Lozano RM Manduch M Tasker RR Kiss ZH Dostrovsky JO Thalamic relay site for cold perception in humans. J Neurophysiol 1999; 81: 1970–3. 10200232 10.1152/jn.1999.81.4.1970

[awv276-B28] Desikan RS Segonne F Fischl B Quinn BT Dickerson BC Blacker D An automated labeling system for subdividing the human cerebral cortex on MRI scans into gyral based regions of interest. Neuroimage 2006; 31: 968–80. 16530430 10.1016/j.neuroimage.2006.01.021

[awv276-B29] Downey LE Fletcher PD Golden HL Mahoney CJ Agustus JL Schott JM Altered body schema processing in frontotemporal dementia with C9ORF72 mutations. J Neurol Neurosurg Psychiatry 2014; 85: 1016–23. 24521566 10.1136/jnnp-2013-306995 PMC4145454

[awv276-B30] Downey LE Mahoney CJ Rossor MN Crutch SJ Warren JD Impaired self-other differentiation in frontotemporal dementia due to the C9ORF72 expansion. Alzheimers Res Ther 2012*;* 4: 42. 23016833 10.1186/alzrt145 PMC3580399

[awv276-B31] Dubois B Feldman HH Jacova C Dekosky ST Barberger-Gateau P Cummings J Research criteria for the diagnosis of Alzheimer's disease: revising the NINCDS-ADRDA criteria. Lancet Neurol 2007; 6: 734–46. 17616482 10.1016/S1474-4422(07)70178-3

[awv276-B32] Erickson JC Clapp LE Ford G Jabbari B Somatosensory auras in refractory temporal lobe epilepsy. Epilepsia 2006; 47: 202–6. 16417550 10.1111/j.1528-1167.2006.00388.x

[awv276-B33] Fletcher PD Downey LE Witoonpanich P Warren JD The brain basis of musicophilia: evidence from frontotemporal lobar degeneration. Front Psychol 2013; 4: 347. 23801975 10.3389/fpsyg.2013.00347 PMC3689257

[awv276-B34] Fletcher PD Downey LE Golden HL Clark CN Slattery CF Paterson RW Auditory hedonic phenotypes in dementia: A behavioural and neuroanatomical analysis. Cortex 2015; 67: 95–105. 25929717 10.1016/j.cortex.2015.03.021 PMC4465962

[awv276-B35] Garibotto V Borroni B Agosti C Premi E Alberici A Eickhoff SB Subcortical and deep cortical atrophy in Frontotemporal Lobar Degeneration. Neurobiol Aging 2011; 32: 875–84. 19501427 10.1016/j.neurobiolaging.2009.05.004

[awv276-B36] Goll JC Crutch SJ Loo JH Rohrer JD Frost C Bamiou DE Non-verbal sound processing in the primary progressive aphasias*.* Brain 2010; 133: 272–85. 19797352 10.1093/brain/awp235 PMC2801322

[awv276-B37] Gorno-Tempini ML Hillis AE Weintraub S Kertesz A Mendez M Cappa SF Classification of primary progressive aphasia and its variants. Neurology 2011; 76: 1006–14. 21325651 10.1212/WNL.0b013e31821103e6 PMC3059138

[awv276-B38] Grecucci A Giorgetta C Bonini N Sanfey AG Reappraising social emotions: the role of inferior frontal gyrus, temporo-parietal junction and insula in interpersonal emotion regulation. Front Hum Neurosci 2013; 7: 523. 24027512 10.3389/fnhum.2013.00523 PMC3759791

[awv276-B39] Greenspan JD Winfield JA Reversible pain and tactile deficits associated with a cerebral tumor compressing the posterior insula and parietal operculum. Pain 1992; 50: 29–39. 1513603 10.1016/0304-3959(92)90109-O

[awv276-B40] Guo CC Gorno-Tempini ML Gesierich B Henry M Trujillo A Shany-Ur T Anterior temporal lobe degeneration produces widespread network-driven dysfunction. Brain 2013; 136: 2979–91. 24072486 10.1093/brain/awt222 PMC3857932

[awv276-B41] Henderson LA Gandevia SC Macefield VG Somatotopic organization of the processing of muscle and cutaneous pain in the left and right insula cortex: a single-trial fMRI study. Pain 2007; 128: 20–30. 17011704 10.1016/j.pain.2006.08.013

[awv276-B42] Henley SM Downey LE Nicholas JM Kinnunen KM Golden HL Buckley A Degradation of cognitive timing mechanisms in behavioural variant frontotemporal dementia. Neuropsychologia 2014; 65: 88–101. 25447066 10.1016/j.neuropsychologia.2014.10.009 PMC4410788

[awv276-B43] Herde L Forster C Strupf M Handwerker HO Itch induced by a novel method leads to limbic deactivations a functional MRI study. J Neurophysiol 2007; 98: 2347–56. 17715198 10.1152/jn.00475.2007

[awv276-B44] Höistad M Barbas H Sequence of information processing for emotions through pathways linking temporal and insular cortices with the amygdala. Neuroimage 2008; 40: 1016–33. 18261932 10.1016/j.neuroimage.2007.12.043 PMC2680198

[awv276-B45] Hsieh S Hornberger M Piguet O Hodges JR Neural basis of music knowledge: evidence from the dementias. Brain 2011; 134: 2523–34. 21857031 10.1093/brain/awr190

[awv276-B46] Irish M Hodges JR Piguet O Right anterior temporal lobe dysfunction underlies theory of mind impairments in semantic dementia. Brain 2014; 137: 1241–53. 24523434 10.1093/brain/awu003

[awv276-B47] Isnard J Magnin M Jung J Mauguiere F Garcia-Larrea L Does the insula tell our brain that we are in pain? Pain 2011; 152: 946–51. 21277680 10.1016/j.pain.2010.12.025

[awv276-B48] Jenkinson M Beckmann CF Behrens TE Woolrich MW Smith SM FSL. Neuroimage 2012; 62: 782–90. 21979382 10.1016/j.neuroimage.2011.09.015

[awv276-B49] Jensen-Dahm C Werner MU Dahl JB Jensen TS Ballegaard M Hejl AM Quantitative sensory testing and pain tolerance in patients with mild to moderate Alzheimer disease compared to healthy control subjects. Pain 2014; 155: 1439–45. 24412285 10.1016/j.pain.2013.12.031

[awv276-B50] Jesso S Morlog D Ross S Pell MD Pasternak SH Mitchell DG The effects of oxytocin on social cognition and behaviour in frontotemporal dementia. Brain 2011; 134: 2493–501. 21859765 10.1093/brain/awr171

[awv276-B51] Kim JH Greenspan JD Coghill RC Ohara S Lenz FA (2007) Lesions limited to the human thalamic principal somatosensory nucleus (ventral caudal) are associated with loss of cold sensations and central pain. J Neurosci 2007; 27: 4995–5004. 17475808 10.1523/JNEUROSCI.0716-07.2007 PMC6672095

[awv276-B52] Kucyi A Hodaie M Davis KD Lateralization in intrinsic functional connectivity of the temporoparietal junction with salience- and attention-related brain networks. J Neurophysiol 2012; 108: 3382–92. 23019004 10.1152/jn.00674.2012

[awv276-B53] Kucyi A Moayedi M Weissman-Fogel I Goldberg MB Freeman BV Tenenbaum HC Enhanced medial prefrontal-default mode network functional connectivity in chronic pain and its association with pain rumination. J Neurosci 2014; 34: 3969–75. 24623774 10.1523/JNEUROSCI.5055-13.2014 PMC6705280

[awv276-B54] Kumfor F Piguet O Disturbance of emotion processing in frontotemporal dementia: a synthesis of cognitive and neuroimaging findings. Neuropsychol Rev 2012; 22: 280–97. 22577002 10.1007/s11065-012-9201-6

[awv276-B55] Landqvist Waldo M Gustafson L Nilsson K Traynor BJ Renton AE Englund E Frontotemporal dementia with a C9ORF72 expansion in a Swedish family: clinical and neuropathological characteristics. Am J Neurodegen Dis 2013; 2: 276–86. PMC3852567 24319645

[awv276-B56] Landqvist Waldo M Santillo AF Gustafson L Englund E Passant U (2014) Somatic complaints in frontotemporal dementia. Am J Neurodegen Dis 2014; 3: 84–92. PMC4162589 25232513

[awv276-B57] Lee SE Khazenzon AM Trujillo AJ Guo CC Yokoyama JS Sha SJ Altered network connectivity in frontotemporal dementia with C9orf72 hexanucleotide repeat expansion. Brain 2014; 137: 3047–60. 25273996 10.1093/brain/awu248 PMC4208465

[awv276-B58] Lenz FA Seike M Richardson RT Lin YC Baker FH Khoja I Thermal and pain sensations evoked by in the area of human ventrocaudal nucleus. J Neurophysiol 1993; 70: 200–12. 8360716 10.1152/jn.1993.70.1.200

[awv276-B59] Letzen JE Craggs JG Perlstein WM Price DD Robinson ME Functional connectivity of the default mode network and its association with pain networks in irritable bowel patients assessed via lidocaine treatment. J Pain 2013; 14: 1077–87. 23743257 10.1016/j.jpain.2013.04.003 PMC3791210

[awv276-B60] Mahoney CJ Beck J Rohrer JD Lashley T Mok K Shakespeare T Frontotemporal dementia with the C9ORF72 hexanucleotide repeat expansion: clinical, neuroanatomical and neuropathological features. Brain 2012; 135: 736–50. 22366791 10.1093/brain/awr361 PMC3286330

[awv276-B61] Masson C Koskas P Cambier J Masson M [Left pseudothalamic cortical syndrome and pain asymbolia]. Rev Neurol 1991; 147: 668–70. 1763257

[awv276-B62] Mazzola L Isnard J Peyron R Guenot M Mauguiere F Somatotopic organization of pain responses to direct electrical stimulation of the human insular cortex. Pain 2009; 146: 99–104. 19665303 10.1016/j.pain.2009.07.014

[awv276-B63] Mazzola L Isnard J Peyron R Mauguiere F Stimulation of the human cortex and the experience of pain: Wilder Penfield's observations revisited. Brain 2012; 135: 631–40. 22036962 10.1093/brain/awr265

[awv276-B64] Meerwijk EL Ford JM Weiss SJ (2013) Brain regions associated with psychological pain: implications for a neural network and its relationship to physical pain. Brain Image Behav 2013; 7: 1–14. 10.1007/s11682-012-9179-y 22660945

[awv276-B65] Menon V Uddin LQ (2010) Saliency, switching, attention and control: a network model of insula function. Brain Struct Funct 2010; 214: 655–67. 20512370 10.1007/s00429-010-0262-0 PMC2899886

[awv276-B66] Mesulam MM From sensation to cognition. Brain 1998; 121: 1013–52. 9648540 10.1093/brain/121.6.1013

[awv276-B67] Moulton EA Becerra L Maleki N Pendse G Tully S Hargreaves R Painful heat reveals hyperexcitability of the temporal pole in interictal and ictal migraine States. Cereb Cortex 2011; 21: 435–48. 20562317 10.1093/cercor/bhq109 PMC3020583

[awv276-B68] Moulton EA Pendse G Becerra, L.R. & Borsook, D. BOLD responses in somatosensory cortices better reflect heat sensation than pain. J Neurosci 2012; 32: 6024–31. 22539862 10.1523/JNEUROSCI.0006-12.2012 PMC3347471

[awv276-B69] Naga AA Devinsky O Barr WB Somatoform disorders after temporal lobectomy. Cogn Behav Neurol 2004; 17: 57–61. 15453513 10.1097/01.wnn.0000117860.44205.78

[awv276-B70] Olausson H Wessberg J Morrison I McGlone F Vallbo A The neurophysiology of unmyelinated tactile afferents. Neurosci Biobehav Rev 2010; 34: 185–91. 18952123 10.1016/j.neubiorev.2008.09.011

[awv276-B71] Omar R Hailstone JC Warren JE Crutch SJ Warren JD The cognitive organization of music knowledge: a clinical analysis. Brain 2010; 133: 1200–13. 20142334 10.1093/brain/awp345 PMC2850578

[awv276-B72] Omar R Henley SM Bartlett JW Hailstone JC Gordon E Sauter DA The structural neuroanatomy of music emotion recognition: evidence from frontotemporal lobar degeneration. Neuroimage 2011a; 56: 1814–21. 21385617 10.1016/j.neuroimage.2011.03.002 PMC3092986

[awv276-B73] Omar R Mahoney CJ Buckley AH Warren JD Flavour identification in frontotemporal lobar degeneration. J Neurol Neurosurg Psychiatry 2013; 84: 88–93. 23138765 10.1136/jnnp-2012-303853 PMC3534254

[awv276-B74] Omar R Rohrer JD Hailstone JC Warren JD Structural neuroanatomy of face processing in frontotemporal lobar degeneration. J Neurol Neurosurg Psychiatry 2011b; 82: 1341–3. 21172863 10.1136/jnnp.2010.227983 PMC3212647

[awv276-B75] Oosterman JM Hendriks H Scott S Lord K White N Sampson EL When pain memories are lost: a pilot study of semantic knowledge of pain in dementia. Pain Med 2014; 15: 751–7. 24401151 10.1111/pme.12336

[awv276-B76] Perry DC Sturm VE Seeley WW Miller BL Kramer JH Rosen HJ Anatomical correlates of reward-seeking behaviours in behavioural variant frontotemporal dementia. Brain 2014; 137: 1621–6. 24740987 10.1093/brain/awu075 PMC4032100

[awv276-B77] Peyron R Laurent B Garcia-Larrea L Functional imaging of brain responses to pain. A review and meta-analysis Neurophysiol Clin 2000; 30: 263–88. 11126640 10.1016/s0987-7053(00)00227-6

[awv276-B78] Pijnenburg YA Gillissen F Jonker C Scheltens P Initial complaints in frontotemporal lobar degeneration. Dem Ger Cogn Dis 2004; 17: 302–6. 10.1159/000077159 15178941

[awv276-B79] Preusser S Thiel SD Rook C Roggenhofer E Kosatschek A Draganski B The perception of touch and the ventral somatosensory pathway. Brain 2015; 138: 540–8. 25541190 10.1093/brain/awu370 PMC4408426

[awv276-B80] Pujol J Macia D Garcia-Fontanals A Blanco-Hinojo L Lopez-Sola M Garcia-Blanco S The contribution of sensory system functional connectivity reduction to clinical pain in fibromyalgia. Pain 2014; 155: 1492–503. 24792477 10.1016/j.pain.2014.04.028

[awv276-B81] Rankin KP Gorno-Tempini ML Allison SC Stanley CM Glenn S Weiner MW Structural anatomy of empathy in neurodegenerative disease. Brain 2006; 129: 2945–56. 17008334 10.1093/brain/awl254 PMC2562652

[awv276-B82] Rascovsky K Hodges JR Knopman D Mendez MF Kramer JH Neuhaus J Sensitivity of revised diagnostic criteria for the behavioural variant of frontotemporal dementia. Brain 2011; 134: 2456–77. 21810890 10.1093/brain/awr179 PMC3170532

[awv276-B83] Ridgway GR Omar R Ourselin S Hill DL Warren JD Fox NC Issues with threshold masking in voxel-based morphometry of atrophied brains. Neuroimage 2009; 44: 99-–111. 18848632 10.1016/j.neuroimage.2008.08.045

[awv276-B85] Rohrer JD Sauter D Scott S Rossor MN Warren JD Receptive prosody in nonfluent primary progressive aphasias. Cortex 2012; 48: 308–16. 21047627 10.1016/j.cortex.2010.09.004 PMC3275751

[awv276-B86] Rohrer JD Nicholas JM Cash DM van Swieten J Dopper E Jiskoot L Presymptomatic cognitive and neuroanatomical changes in genetic frontotemporal dementia in the Genetic Frontotemporal dementia Initiative (GENFI) study: a cross-sectional analysis. Lancet Neurol 2015; 14: 253–62. 25662776 10.1016/S1474-4422(14)70324-2 PMC6742501

[awv276-B87] Schmahmann JD Leifer D Parietal pseudothalamic pain syndrome. Clinical features and anatomic correlates. Arch Neurol 1992; 49: 1032–7. 1417510 10.1001/archneur.1992.00530340048017

[awv276-B88] Seeley WW Allman JM Carlin DA Crawford RK Macedo MN Greicius MD Divergent social functioning in behavioral variant frontotemporal dementia and Alzheimer disease: reciprocal networks and neuronal evolution. Alzheimers Dis Assoc Disord 2007a; 21: S50–7. 10.1097/WAD.0b013e31815c0f14 18090425

[awv276-B89] Seeley WW Crawford RK Zhou J Miller BL Greicius MD Neurodegenerative diseases target large-scale human brain networks. Neuron 2009; 62: 42–52. 19376066 10.1016/j.neuron.2009.03.024 PMC2691647

[awv276-B90] Seeley WW Menon V Schatzberg AF Keller J Glover GH Kenna H Dissociable intrinsic connectivity networks for salience processing and executive control. J Neurosci 2007b; 27: 2349–56. 17329432 10.1523/JNEUROSCI.5587-06.2007 PMC2680293

[awv276-B91] Shipp S The functional logic of cortico-pulvinar connections. Phil Trans R Soc Lond B Biol Sci 2003; 35: 1605–24. 14561322 10.1098/rstb.2002.1213 PMC1693262

[awv276-B92] Singer T Seymour B O'Doherty J Kaube H Dolan RJ Frith CD Empathy for pain involves the affective but not sensory components of pain. Science 2004; 303: 1157–62. 14976305 10.1126/science.1093535

[awv276-B93] Snowden JS Bathgate D Varma A Blackshaw A Gibbons ZC Neary D Distinct behavioural profiles in frontotemporal dementia and semantic dementia. J Neurol Neurosurg Psychiatry 2001; 70: 323–32. 11181853 10.1136/jnnp.70.3.323 PMC1737271

[awv276-B94] Snowden JS Harris J Richardson A Rollinson S Thompson JC Neary D Frontotemporal dementia with amyotrophic lateral sclerosis: a clinical comparison of patients with and without repeat expansions in C9orf72. Amyotroph Lateral Scler Frontotemporal Degener 2013; 14: 172–76. 23421625 10.3109/21678421.2013.765485

[awv276-B95] Snowden JS Rollinson S Thompson JC Harris JM Stopford CL Richardson AMT Distinct clinical and pathological characteristics of frontotemporal dementia associated with C9ORF72 mutations. Brain 2012; 135: 693–708. 22300873 10.1093/brain/awr355 PMC3286329

[awv276-B96] Sprenger T Seifert CL Valet M Andreou AP Foerschler A Zimmer C Assessing the risk of central post-stroke pain of thalamic origin by lesion mapping. Brain 2012; 135: 2536–45. 22719000 10.1093/brain/aws153

[awv276-B97] Sridharan D Levitin DJ Menon V A critical role for the right fronto-insular cortex in switching between central-executive and default-mode networks. Proc Natl Acad Sci USA 2008; 105: 12569–74. 18723676 10.1073/pnas.0800005105 PMC2527952

[awv276-B98] Sturm VE Yokoyama JS Seeley WW Kramer JH Miller BL Rankin KP Heightened emotional contagion in mild cognitive impairment and Alzheimer's disease is associated with temporal lobe degeneration. Proc Natl Acad Sci USA 2013; 110: 9944–9. 23716653 10.1073/pnas.1301119110 PMC3683715

[awv276-B99] Takada LT Sha SJ Neuropsychiatric features of C9orf72-associated behavioral variant frontotemporal dementia and frontotemporal dementia with motor neuron disease. Alzheimers Res Ther 2012; 4: 38. 23034079 10.1186/alzrt141 PMC3580395

[awv276-B100] Warren JD Fletcher PD Golden HL The paradox of syndromic diversity in Alzheimer disease. Nat Rev Neurol 2012; 8: 451–64. 22801974 10.1038/nrneurol.2012.135

[awv276-B101] Whitwell JL Sampson EL Loy CT Warren JE Rossor MN Fox NC VBM signatures of abnormal eating behaviours in frontotemporal lobar degeneration. Neuroimage 2007; 35: 207–13. 17240166 10.1016/j.neuroimage.2006.12.006

[awv276-B102] Whitwell JL Weigand SD Boeve BF Senjem ML Gunter JL DeJesus-Hernandez M Neuroimaging signatures of frontotemporal dementia genetics: C9ORF72, tau, progranulin and sporadics. Brain 2012; 135: 794–806. 22366795 10.1093/brain/aws001 PMC3286334

[awv276-B103] Wiener M Coslett HB Disruption of temporal processing in a subject with probable frontotemporal dementia. Neuropsychologia 2008; 46: 1927–39. 18329055 10.1016/j.neuropsychologia.2008.01.021 PMC2494711

[awv276-B104] Woolley JD Khan BK Natesan A Karydas A Dallman M Havel P Satiety-related hormonal dysregulation in behavioral variant frontotemporal dementia. Neurology 2014; 82: 512–20. 24415571 10.1212/WNL.0000000000000106 PMC3937860

[awv276-B105] Zahn R Moll J Iyengar V Huey ED Tierney M Krueger F Social conceptual impairments in frontotemporal lobar degeneration with right anterior temporal hypometabolism. Brain 2009; 132: 604–16. 19153155 10.1093/brain/awn343 PMC2724922

[awv276-B106] Zhou J Greicius MD Gennatas ED Growdon ME Jang JY Rabinovici GD Divergent network connectivity changes in behavioural variant frontotemporal dementia and Alzheimer's disease. Brain 2010; 133: 1352–67. 20410145 10.1093/brain/awq075 PMC2912696

[awv276-B107] Zhou J Seeley WW Network dysfunction in Alzheimer's disease and frontotemporal dementia: implications for psychiatry. Biol Psychiatry 2014; 75: 565–73. 24629669 10.1016/j.biopsych.2014.01.020

